# Violation of lockdown norms and peaks in daily number of positive cases to COVID-19 in Italy

**DOI:** 10.35241/emeraldopenres.13699.1

**Published:** 2020-05-13

**Authors:** Gabriele Ruiu, Maria Laura Ruiu

**Affiliations:** 1 Department of Economics and Business, University of Sassari, Sassari, Italy; 2 Department of Social Science, Northumbria University, Newcastle Upon Tyne, UK

**Keywords:** Covid19, Lockdown, Italy, Contagion

## Abstract

Italy has been the first Western Country to suffer a massive outbreak of COVID-19. Starting from the 11 ^st^ of March 2020, the Italian Government approved a series of emergency restrictive measures to limit people’s movement and social contacts. The aim of this short paper is to test if the number of norm-violations (related to people’s movement) might contribute to the peaks of new COVID-19 positives after few days. We show that peaks in the violations of the lockdown norms correspond to peaks in new positive cases about 6 days later.

## Introduction

**Figure 1.**
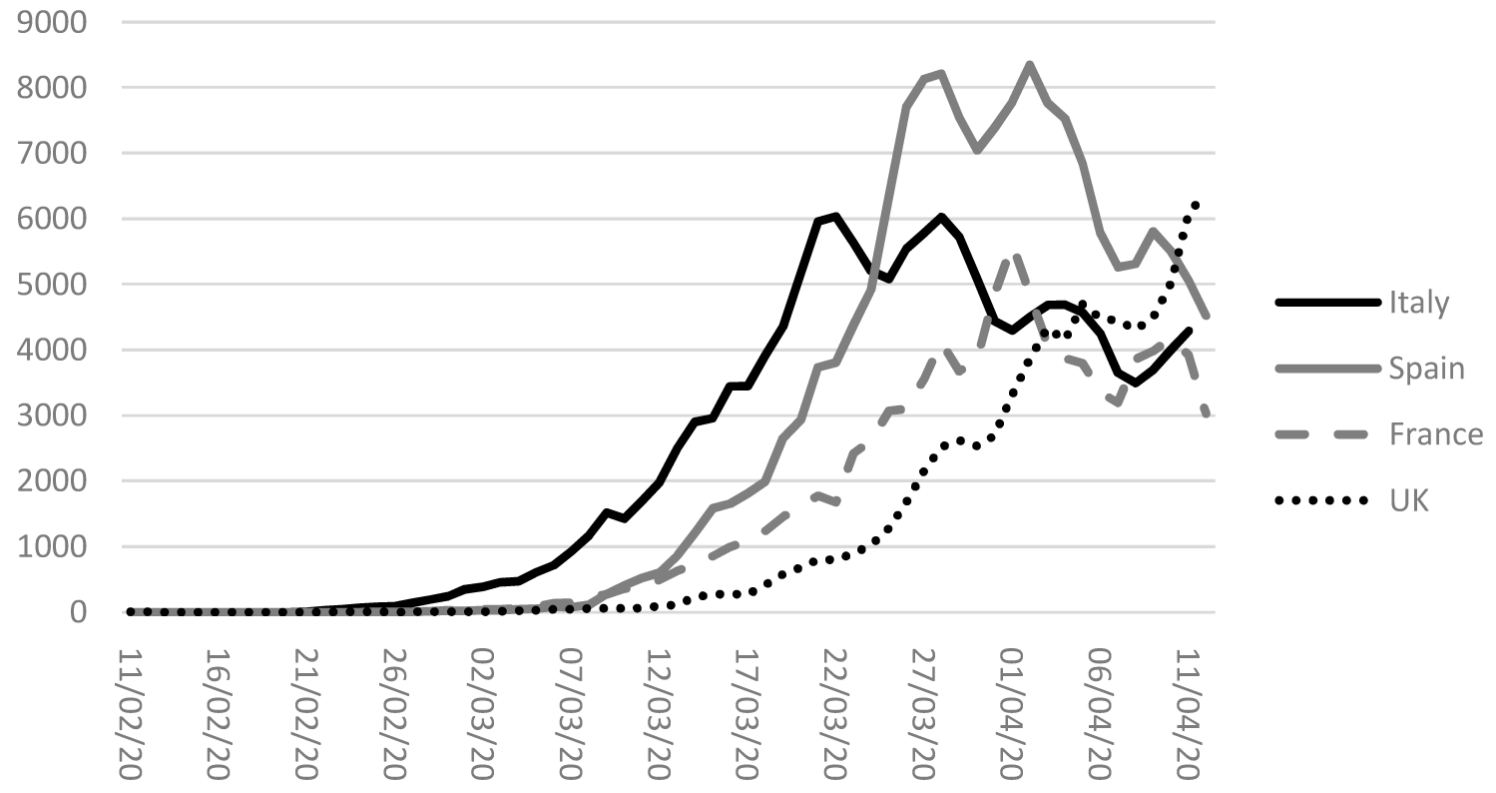
Coronavirus disease 2019 (COVID-19), daily new confirmed case, rolling 3-day average. Source: https://ourworldindata.org/coronavirus#covid-19-cases-by-country.

In three months, after the first cases of coronavirus disease 2019 (COVID-19) in China ( Corman *et al.*, 2020) and the identification of a novel virus on the 7 ^th^ of January 2020 ( WHO, 2020a), the Covid-19 outbreak was classified as a global threat and declared a pandemic on the 11 ^th^ of March ( WHO, 2020b). Italy has been the first Western Country to suffer a large outbreak of COVID-19 (see Figure 1). To contain the spread of the virus, the Italian Government approved a series of emergency restrictive measures to limit people’s movement and social contacts. Between February 21 and 22, 11 municipalities in Northern Italy were declared locked down - people were not allowed to enter or leave the affected areas. On February 25, schools, universities and public offices were closed in six out of seven Northern regions. On the 4 ^rth^ of March these restrictions were extended to the entire Italian territory. On the 8 ^th^ of March the Lombardy region and additional 14 Northern provinces were locked down ( Gazzetta Ufficiale, 2020a).

These dispositions involved about 16 million individuals. Finally, on the 10 ^th^ of March the lockdown was extended to the entire country ( Gazzetta Ufficiale, 2020b). The lockdown imposed the closure of non-essential commercial businesses (restaurants, pubs, libraries, etc.), banned people gatherings, and restricted people’s movement (only for strictly necessary needs such as e.g. food, essential work and health-related reasons). Obviously, essential commercial activities (e.g. groceries and pharmacies) were urged to adopt rigid norms to ensure physical distance. Several times, the management of the outbreak was undermined by the spread of fake news, leak of decrees’ drafts and political rivalry. For example, a leak of the information contained in a decree draft ( Gazzetta Ufficiale, 2020a), relative to the imminent lock down of Lombardy (and other 14 provinces), caused panic and confusion in the public understanding of the events. In fact, thousands of people fled from the North to the South of Italy.

This event forced the government to extend the lockdown to the entire country three days later. Severe fines (and imprisonment for people positive to the virus) for anyone leaving home unauthorised were established. Hence, starting from the 11 ^st^ of March 2020, the Italian Ministry of the Interior has been updating data on the daily number of both controls and fines due to the violation of the lockdown norms ( Ministero dell’Interno, 2020). Figure 1 shows the number of daily new cases of Covid-19 in the most affected countries worldwide (during the first phases of the pandemic). These countries show peaks and troughs in the number of cases during both the increasing and decreasing trends. Therefore, the aim of this short paper is to test if the number of norm-violations (related to people’s movement) might contribute to the peaks of new Covid-19 positives after a few days. The first section illustrates data and methodology; the second section shows and comments the results of the analysis; the last section is devoted to final considerations.

## Methods

To explore the relationship between the violation of lockdown restrictions and the spread of COVID-19, we estimate the following log-linear regression model:

$$\left(\text{1}\right)\;\text{log}\left(p_{t}\right)=\beta_{0}+\sum_{j=1}^{j=7}\beta_{j}d_{j}+\beta_{8}time+\beta_{9}time^{2}+\beta_{10}Sanction\_rate_{t-k}+\beta_{11}n\_test_{t}+\varepsilon_{t}$$

Where p is the daily count of new positive cases, t=17/03,…, 20/04, d is a dummy variable for each day of the weak (1 =Monday, 2= Thursday, …, 7 = Sunday), while with “time” and “time ^2^” we allow for the presence of a quadratic trend in the time series (as suggested by the shape of the Italian curve in Figure 1). ε is a random error. The “sanction rate” is calculated as the ratio between the daily number of fines and the number of checks carried out by the Police. This ratio was then multiplied by 100 to interpret it as a percentage. The sanction rate is used in this paper as an approximation of the level of disrespect of the COVID-19 restrictions. Obviously, this represents only a proxy given that not all the individuals that have violated the dispositions have been caught by police authorities. k is set equal to 6 and it has been selected looking at the cross-correlation function between the sanction rate and the daily new positive cases ^1^. Finally, n_test if the number of COVID-19 tests implemented in day t. Alternatively, we also run a negative binomial regression using the same dependent and independent variables to take into account the discrete nature of the dependent variable (the results section shows that a negative binomial regression better fits the data compared to a poisson regression due to over-dispersion). ^2^.

The sanction rate is based on the data provided by the Italian Ministry of the Interior, whereas the daily count of new positive cases and the count of COVID-19 tests are provided by the Italian Civil Protection ( Dipartimento della Protezione Civile, 2020). All these data are publicly available. The data on the sanctions for the violation of lockdown norms could be downloaded from https://www.interno.gov.it/it/coronavirus-i-dati-dei-servizi-controllo. Unfortunately. the website is written only in Italian language. The data on daily positive cases could be downloaded from http://opendatadpc.maps.arcgis.com/apps/opsdashboard/index.html#/b0c68bce2cce478eaac82fe38d4138b1.

All the statistical analyses presented in this paper have been carried out using Stata 16. Alternatively, these analyses could be easily carried out in open access software as R version 3.5.2 (or later versions).

## Results

**Figure 2.**
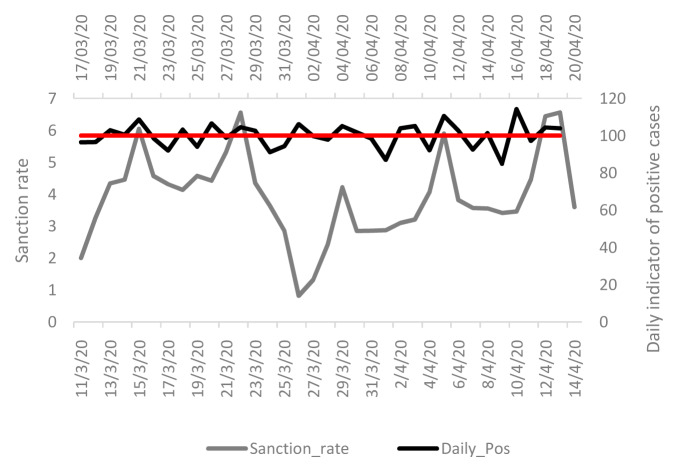
Sanction rates and daily de-trended indicator of new positive cases.

As initial descriptive evidence, Figure 2 ( Ruiu & Ruiu, 2020) shows the daily sanction rate registered in Italy from the 11 ^th^ of March (official lockdown of the country) to the 14 ^th^ of April (two days after Easter), and the associated daily de-trended indicator of new positive cases (taken 6 days after the sanctions).

In particular, the latter indicator has been obtained by dividing the observed number of new cases in each day for a three day moving average (the result has been multiplied by 100). When the de-trended indicator is above/below 100, it indicates that the daily number of positive cases is above/below the moving average. The red line is plotted as a reference. Peaks in the sanction rate seem to coincide with peaks in the daily indicator of positive cases. Note also that peaks in the sanction rate correspond to weekends. The latter evidence may be due to the difficulty of people to renounce the habit of going out during spring weekends, even during the COVID-19 pandemic. Note also that on the 16 ^th^ April a peak in positive cases can be observed, and that 6 days before it was Good Friday, which is the first day of the Catholic Easter Holiday. However, the increase in norm violations due to the holiday period seems not to be caught by the sanction rates series.

Table 1 (a,b) ( Ruiu & Ruiu, 2020) shows the results of the ordinary least squares (OLS) estimation of equation 1 and of the negative binomial regression (NBR), respectively. The result of the over-dispersion test suggested that a negative binomial regression better fitted the data compared to a poisson regression. The bottom of the table reports the Breusch-Godfrey auto-correlation test carried out to the model estimated in column a, the Breusch-Pagan test for heteroskedasticity and the Shapiro-Wilk test for the normality, the over-dispersion test that led to the use of a negative binomial regression model.

**Table 1.** Effect of violation of lockdown norms on daily positive cases. Italy, 17 March 2020- 20 April 2020.

	(a) OLS Log(p)	(b) NBR p
Sanction_rate(-6)	0.046 ^***^ (0.016)	0.045 ^***^ (0.013)
time	0.021 ^**^ (0.009)	0.021 ^***^ (0.008)
time ^2^	-0.001 ^***^ (0.000)	-0.001 ^***^ (0.000)
**Day of the week**		
Monday	-0.045 (0.060)	-0.044 (0.049)
Tuesday	-0.176 ^**^ (0.070)	-0.176 ^***^ (0.058)
Wednesday	-0.211 ^***^ (0.066)	-0.205 ^***^ (0.055)
Thursday	-0.146 ^**^ (0.053)	-0.141 ^***^ (0.044)
Friday	Ref	ref
Saturday	-0.041 (0.053)	-0.040 (0.044)
Sunday	-0.034 (0.065)	-0.032 (0.053)
n_test	0.000 (0.000)	0.000* (0.000)
_cons	8.228 ^***^ (0.126)	8.232 ^***^ (0.106)
Over-dispersion parameter		-5.451 ^***^ (0.253)
N	35	35
R2/ McFadden’s Pseudo R2	0.90	0.156
Breusch Godfrey 1° order autocorrelation test	Chi2: 0.616;	p-value: 0.43
Breusch Pagan test	Chi2: 1.43;	p-value: 0.23
Shapiro-Wilk Normality test	W: 0.958;	p-value: 0.207
Over dispersion test	Chi2: 649.83;	p-value: 0.000

OLS - ordinary least squares, NBR - negative binomial regression Standard errors in parentheses * *p* < 0.10, ** *p* < 0.05, *** *p* < 0.01 Note: n_test is the number of COVID-19 tests carried out each day._const is the constant of the linear model. time and time ^2^ capture the quadratic trend in the evolution of contagions. Sanction_rate(-6) is the the ratio between the daily number of fines for the violation of lockdown norms and the number of checks carried out by the Italian Police. This variable is measured six days before the number of contagions.

The results reported in column (a) suggest that an increase of one-point percentage in the sanction rate corresponds to about a 4.6% increase in the number of positive cases 6 days later (the result is very similar in the case of negative binomial regression). This effect should be taken into account since the sanction rate varies from a minimum of about 1% on Thursday to a maximum of about 6% in the weekend. This means that in general 6 days after a weekend we observe about 20% more positive cases. The result is statistically significant at the 1% level (p-value <0.01). Note also that since the violations of lockdown norms are only proxied by the sanctions, we have a case of measurement error in the independent variable. According to Wooldridge (2002) when the Beta parameter of the OLS regression is positive, we have an attenuation bias in the estimated parameter. In other words, this means that the effect of the violation of lockdown norms is even larger.

**Figure 3.**
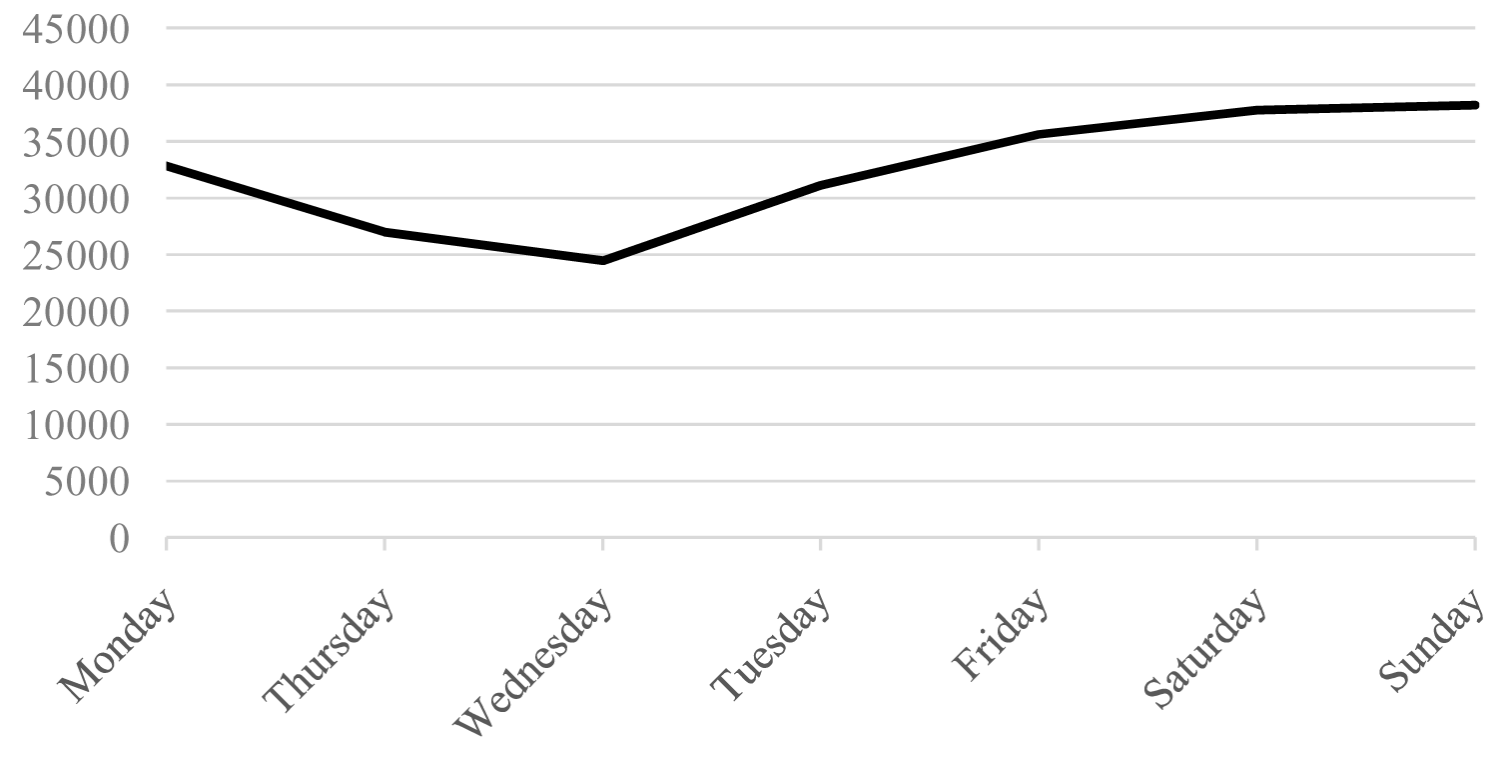
Average number of tests carried out in each day of the week from 11 March to 20 April.

Note that the number of COVID-19 tests is not statistically significant in column (a), while it is only weakly significant in column (b). This means that the observed variations in the curve that represents the daily positive cases do not depend on the number of tests carried out. However, it must be also noted that the effects of the daily variation in the number of tests might be partially captured to by the set of dummies related to the day of the week . In particular, tests seem to be concentrated in the second part of the week (see Figure 3 ( Ruiu & Ruiu, 2020)). In any case, the estimated effect of the lockdown violation is the estimated effect after having controlled for the latter source of daily variation.

Finally, note that the diagnostic tests reported at the bottom of Table 1 ( Ruiu & Ruiu, 2020) suggests that the hypotheses of homoskedasticity, absence of autocorrelation and normality cannot be rejected.

## Discussion and conclusion

Recently in Italy, as well as in other countries, protests against lockdown restrictions have been increasing. The debate has been animated by an article ( Comelli, 2020) that appeared in one of the most read Italian newspaper, *Il Corriere della Sera*, according to which COVID-19 spread was influenced by the lockdown only in the first 17 days. The key message of this article is that in Italy the number of cases was mainly driven by the initial distribution of cases. Therefore, after the 17 ^th^ day the measures have become ineffective and must be abandoned to limit further economic damages. However, this conclusion was drawn upon an incorrect interpretation of a mathematical model used to project the number of contagions. In fact, the impact of a policy should be evaluated through a counterfactual analysis, which is a comparison between what happened and what would have happened without intervention ( Mahoney & Barrenechea, 2019). The predictions of the mathematical model cited in this newspaper article were indeed based on the observation of the contagion dynamics during the first 17 days of lockdown. Therefore, these considerations were exclusively based on factual analysis without considering counterfactual conditionals. By contrast, we showed a relation between the violation of lockdown norms and peaks in the daily number of new positive cases. Using the number of fines issued for coronavirus lockdown breaches, we estimated that an increase of one-point percentage in the sanction rate resulted in an increase of about 4.6% in the number of positive cases six days later. This result is further supported by modelling studies conducted in China, which shows how the premature lifting of measures might produce an earlier second peak ( Leung *et al.*, 2020; Prem *et al.*, 2020).

Some lessons can be learned from the implementation of COVID-19 restrictions in Italy. First, our results suggest that in the absence of pharmaceutical treatments, restrictions are essential to contain the spread of the virus and avoid overwhelming the health-care system ( Colbourn, 2020; Xu & Li, 2020). This is further supported by the effects produced by the management system adopted by both China ( Leung *et al.*, 2020) and Singapore ( Koo *et al.*, 2020; Lewnard & Lo, 2020). Both cases show that easing restrictions when the number of affected individuals is relatively small would cause an exponential increase of cases. Physical distancing, quarantining infected individuals and their direct contacts, and closure of schools and public services were also shown to be effective in previous outbreaks (e.g. SARS) ( James *et al.*, 2006). Therefore, governmental management seems to be critical in controlling the spread of the virus until either a vaccine or effective treatment is developed ( Tan, 2006).

Second, this paper suggests the necessity to efficiently communicate the risks related to a disrespect of the rules. The need for imposing fines (and the official number of breaches to the norms) suggests a government-citizen miscommunication. The initial chaos generated by the emergence of a novel pathogen caused difficulty in both developing (from a government perspective) and adapting (from a citizen perspective) to the restrictions during the crisis. Shortfalls in preparedness, plus an initial underestimation of the problem by the population, followed by panic reactions (e.g. massive assault to train stations to flee from the North to the South), caused a delay in containing the spread of the virus. However, after an initial period of chaos, the set of restrictions established by the Government (informed by the Chinese management model) started to produce some results in terms of supressing and reducing the number of new cases. While an article published by the the *New York Times* attributed the initial Italian mismanagement of the crisis to an innate attitude of Italian people to break the rules ( Horowitz & Bubola, 2020), an alternative explanation should be traced back given the lack of a clear plan for disaster managing ( Houston *et al.*, 2014).

This point is directly connected to the third lesson learned that is the necessity for national preparedness for future outbreaks ( SARS Investigation Team, 2005). In fact, previous cases (see the SARS outbreak) show that early identification and immediate intervention (such as e.g. quarantine of affected and their families) are fundamental to control the spread of an emerging pathogen ( Centers for Disease Control and Prevention, 2003). After the influenza AH1N1 in 2009, the WHO, (2013), WHO, (2017) produced some guidelines to manage pandemic influenza-related risks. These plans contained guidelines to harmonise national responses in the case of a pandemic. One of the key points throughout the pandemic (pre, during and post-crisis phases) is represented by the efficient communication and dissemination of information and actions needed to prepare the population to deal with the crisis. The increase in the number of fines during weekends in Italy, shows that the government was not able to efficiently prepare citizens to accept the lockdown restrictions ( Ruiu, 2020). Therefore, despite the existence of a national plan ( Italian Government, 2017), developed in accordance to the WHO recommendations, the management of the crisis encountered some difficulties. Future research should look at the potential reason behind this misalignment between global, national and local levels.

## Data availability

### Source data

Sanctions for the violation of lockdown norms: https://www.interno.gov.it/it/coronavirus-i-dati-dei-servizi-controllo.

The data on daily positive cases:

http://opendatadpc.maps.arcgis.com/apps/opsdashboard/index.html#/b0c68bce2cce478eaac82fe38d4138b1.

### Underlying data

Harvard Dataverse: Ruiu_Ruiu_ViolationOfLockdownNorms. https://doi.org/10.7910/DVN/WCRTS3 ( Ruiu & Ruiu, 2020).

This project contains the following underlying data:

Ruiu_Ruiu_ViolationLockdownandPositive.tab (text file containing the data used in this paper. Note that the first row contains variable names and the column separator is the semicolon).

Dataset_Description.txt (text file containing detailed information on each variable included in the dataset Ruiu_Ruiu_ViolationLockdownandPositive.tab).

Data are available under the terms of the Creative Commons Zero “No rights reserved” data waiver (CC0 1.0 Public domain dedication).

## Notes

^1^ The cross covariance function of lag k between two time series X and Y may be defined as R _yx_(k)= COV(X _t_; Y _t+k_), where t is time and k=[-Q,..,0,..+Q], the function of cross covariance is not symmetic around lag 0, i.e. R _yx_(k) ≠ R _yx_(-k). Thus, the cross correlation is given by $\rho_{yx}(k)=\frac{R_{yx}(k)}{\sqrt{\sigma_{y}^{2}\sigma_{x}^{2}}}.$ The World Health Organization reports that “Most estimates of the incubation period for COVID-19 range from 1-14 days, most commonly around five days”. Then, Q was equal to 14, looking at the cross correlation function between the two time series, the maximum value of the coefficient of crosscorrelation was found after 6 days.
