# Supplementary material for: Violation of lockdown norms and peaks in daily number of positive cases to COVID-19 in Italy
Source: Emerald Open Res. 2020 May 13;2:25. doi: 10.35241/emeraldopenres.13699.1 (PMC7313553; doi:10.35241/emeraldopenres.13699.1)
Supplement: Peer review history

# Peer review: Reviewer response for version 1

**DOI:** 10.21956/emeraldopenres.14773.r26816

**Published:** 2020-06-09

**Authors:** Josep Bernabeu-Mestre

**Affiliations:** 1 Department of Community Nursing, Preventive Medicine and Public Health and History of science, Faculty of Health Sciences, University of Alicante, Alicante, Spain

Based on the Italian case, the authors propose an original and interesting investigation for the current epidemiological context.

 The work raises a clear and defined objective and through an adequate methodology it obtains results that allow answering the question posed.

 The applicability of the results obtained and their interest in designing future strategies against epidemics such as COVID-19 deserve to be highlighted.

 One of the most outstanding and applicable conclusions of the work, is the need to involve citizens in the measures adopted, through awareness campaigns. This would lead to greater collaboration on the part of citizens and avoid punitive measures such as those that are studied at work. Although it is a case study, the working hypothesis and the methodology used, will allow similar research in other contexts. The comparison of the results obtained could serve to relate the degree of monitoring and compliance with the type of containment measures applied.

Is the work clearly and accurately presented and does it cite the current literature?

Yes

If applicable, is the statistical analysis and its interpretation appropriate?

Yes

Are all the source data underlying the results available to ensure full reproducibility?

Yes

Is the study design appropriate and is the work technically sound?

Yes

Are the conclusions drawn adequately supported by the results?

Yes

Are sufficient details of methods and analysis provided to allow replication by others?

Yes

Reviewer Expertise:

Historical epidemiology and health policies

I confirm that I have read this submission and believe that I have an appropriate level of expertise to confirm that it is of an acceptable scientific standard.

# Response

**Published:** 2020-06-09

**Authors:** Gabriele Ruiu

**Affiliations:** University of Sassari, Italy

Thank you very much for the appreciation of our work.

 Best regards,

 The authors

# Peer review: Reviewer response for version 1

**DOI:** 10.21956/emeraldopenres.14773.r26812

**Published:** 2020-05-15

**Authors:** Barbara Backstrom

**Affiliations:** 1 Social Sciences and Management Department, Universidade Aberta (UAb), Lisbon, Portugal

The work clearly and accurately presented the current literature.

 The study design is appropriate and there are sufficient details of methods and analysis provided to allow replication by others.

 A qualified statistician is required to evaluate the statistical analysis.

 The source data underlying the results available ensure reproducibility.

 The conclusions drawn are adequately supported by the results.

Is the work clearly and accurately presented and does it cite the current literature?

Yes

If applicable, is the statistical analysis and its interpretation appropriate?

I cannot comment. A qualified statistician is required.

Are all the source data underlying the results available to ensure full reproducibility?

Yes

Is the study design appropriate and is the work technically sound?

Yes

Are the conclusions drawn adequately supported by the results?

Yes

Are sufficient details of methods and analysis provided to allow replication by others?

Yes

Reviewer Expertise:

sociology, demography

I confirm that I have read this submission and believe that I have an appropriate level of expertise to confirm that it is of an acceptable scientific standard.

# Response

**Published:** 2020-05-15

**Authors:** Gabriele Ruiu

**Affiliations:** University of Sassari, Italy

Dear Barbara,

Thank you very much for the appreciation of our work. 

Best regards,

Gabriele and Maria Laura Ruiu
